# Health Tract, No. 218

**Published:** 1865

**Authors:** 


### DIGESTIBILITY OF FOOD.

THE following table of the digestibility of the most common articles of food prepared from standard authorities, is approximately correct and is of very general practical interest:

| *Quality.* | *Preparation.* | *Time of Digestion.* H. M. |
|---|---|---|
| Rice | Boiled, | 1 00 |
| Pigs' feet, soused | " | 1 00 |
| Tripe, soused | " | 1 00 |
| Eggs, whipped | Raw, | 1 30 |
| Trout, salmon, fresh | Boiled, | 1 30 |
| Trout, salmon, fresh | Fried, | 1 30 |
| Soup, barley | Boiled, | 1 30 |
| Apples, sweet, mellow | Raw, | 1 30 |
| Venison steak | Broiled, | 1 35 |
| Brains, animal | Boiled, | 1 45 |
| Sago | " | 1 45 |
| Tapioca | " | 2 00 |
| Barley | " | 2 00 |
| Milk | " | 2 00 |
| Liver, beef's, fresh | Broiled, | 2 00 |
| Eggs, fresh | Raw, | 2 00 |
| Codfish, cured dry | Boiled, | 2 00 |
| Apples, sour, mellow | Raw, | 2 00 |
| Cabbage, with vinegar | " | 2 00 |
| Milk | " | 2 15 |
| Eggs, fresh | Roasted, | 2 15 |
| Turkey, wild | " | 2 18 |
| Turkey, domestic | Boiled, | 2 25 |
| Gelatine | " | 2 30 |
| Turkey, domestic | Roasted, | 2 30 |
| Goose, wild | " | 2 30 |
| Pig, sucking | " | 2 30 |
| Lamb, fresh | Broiled, | 2 30 |
| Hash, meat, and vegetables | Warmed, | 2 30 |
| Beans, pod | Boiled, | 2 30 |
| Cake, sponge | Baked, | 2 30 |
| Parsnips | Boiled, | 2 30 |
| Potatoes, Irish | Roasted, | 2 30 |
| Cabbage, head | Raw, | 2 30 |
| Spinal marrow, animal | Boiled, | 2 40 |
| Chicken, full grown | Fricasseed, | 2 45 |
| Custard | Baked, | 2 45 |
| Beef, with salt only | Boiled, | 2 45 |
| Apples, sour, hard | Raw, | 2 50 |
| Oysters, fresh | " | 2 55 |
| Eggs, fresh | Soft boiled, | 3 00 |
| Bass, striped, fresh | Broiled, | 3 00 |
| Beef, fresh, lean, rare | Roasted, | 3 00 |
| Pork, recently salted | Stewed, | 3 00 |
| Mutton, fresh | Broiled, | 3 00 |
| Soup | Boiled, | 3 00 |
| Chicken soup | " | 3 00 |
| Aponeurosis | " | 3 00 |
| Dumpllng, apple | " | 3 00 |
| Cake, corn | Baked, | 3 00 |
| Oysters, fresh | Roasted, | 3 15 |
| Porksteak | Broiled, | 3 15 |
| Mutton, fresh | Roasted, | 3 15 |
| Bread, corn | Baked, | 3 15 |
| Carrot, orange | Boiled, | 3 15 |
| Sausage, fresh | Broiled, | 3 30 |
| Flounder, fresh | Fried, | 3 30 |
| Catfish, fresh | " | 3 30 |
| Oysters, fresh | Stewed, | 3 30 |
| Butter | Melted, | 3 30 |
| Cheese, old, strong | Raw, | 3 30 |
| Soup, mutton | Boiled, | 3 30 |
| Oyster soup | " | 3 03 |
| Bread, wheat, fresh | Baked, | 3 30 |
| Turnips, flat | Boiled, | 3 30 |
| Potatoes, Irish | " | 3 30 |
| Eggs, fresh | Hard boiled, | 3 30 |
| Green corn and beans | Boiled, | 3 45 |
| Beets | " | 3 45 |
| Salmon, salted | " | 4 00 |
| Beef | Fried, | 4 00 |
| Veal, fresh | Broiled, | 4 00 |
| Fowls, domestic | Roasted, | 4 00 |
| Soup, beef, vegetables, and bread | Boiled, | 4 00 |
| Heart, animal | Fried, | 4 00 |
| Beef, old, hard, salted | Boiled, | 4 15 |
| Soup, marrow-bones | " | 4 15 |
| Cartilage | " | 4 15 |
| Pork, recently salted | " | 4 30 |
| Veal, fresh | Fried, | 4 30 |
| Ducks, wild | Roasted, | 4 30 |
| Suet, mutton | Boiled, | 4 30 |
| Cabbage | " | 4 30 |
| Pork, fat and lean | Roasted, | 5 15 |
| Tendon | Boiled, | 5 30 |
| Suet, beef, fresh | " | 5 30 |

